# Investigating metalinguistic written corrective feedback focused on EFL learners’ discourse markers accuracy in mobile-mediated context

**DOI:** 10.1186/s40862-021-00111-8

**Published:** 2021-01-25

**Authors:** Natasha Pourdana, Payam Nour, Fariba Yousefi

**Affiliations:** 1 Department of Teaching English and Translation, Faculty of Literature and Foreign Languages, Karaj Branch, Islamic Azad University, Karaj, Iran; 2 Department of English Language, Faculty of Literature and Humanities, Imam Khomeini International University, Qazvin, Iran

**Keywords:** Corrective, Discourse markers, Feedback, Metalinguistic, Mobile-mediated

## Abstract

Among a growing body of research that examined the contradictory role of written corrective feedback (WCF) in development of L2 writing accuracy, this study investigated the possible impact of focused metalinguistic WCF on discourse markers (DMs) in writing performance of an intact group of 42 Iranian English as a Foreign Language (EFL) learners over an eight-week period. In an authentic, situated, and personalized learning platform, giving and receiving WCF were made possible only through the mobile-mediated application of WhatsApp. Before participants wrote on selected elicitation topics, they had taken part in a 2004 version of Oxford Preliminary Test in order to be screened for their initial differences in writing performance. After receiving metalinguistic WCF on their scripts, participants were required to work on the coded feedback and try to eliminate the DM errors in their revised writing assignment. After collecting the scripts over an eight-week period, the content of written assignment was thematically analyzed using NVivo 21 Software for the additive, adversative, causal and temporal DMs, following Halliday and Hasan’s (Cohesion in English., 1976) typology. In a convergent mixed-methods design, the content analysis of the qualitative data showed a larger distribution of additive DM than adversative, causal, and temporal DMs in all participants’ written scripts. Exploring the possible impact of metalinguistic WCF on improving the DMs accuracy, analysis of the frequency count data with Statistical Package for the Social Sciences (SPSS) multivariate Chi-square test reported the fluctuation and unsystematic patterns of distribution for four types of DMs with no sign of significant long-term improvement in DMs accuracy after receiving metalinguistic WCF. These findings implied further research on practicing alternative WCF strategies focused on variety of error types in actual and virtual L2 writing environments.

## Introduction

Proliferation of research in favor of written corrective feedback (WCF) in second/foreign language (L2) contexts does not protect any language teachers from losing sight of how to interpret the contradictory research findings and how to take actions to apply them in real classroom context. For many L2 teaching professionals, the critical concern about WCF has never been whether it is useful, but *how* and *when* to use it effectively to help students improve their writing performance. Many studies verified the viability of WCF in improving L2 learners writing (e.g., Ellis, Loewen, & Erlam, 2006; Farrokhi, Zohrabi, & Chehr Azad, 2017; Ferris, 2006; Gholami & Narimani, 2012; Hashemian & Farhang-ju, 2018; Li, 2010; Pourmousavi & Mohamadi Zenouzagh, 2020). Likewise, the growing supports indicated that WCF can notably draw the language learners’ attention to accurate linguistic forms that plays a critical role in facilitating L2 language acquisition (e.g., Gu & Wang, 2008; Hino, 2006; Iwashita, 2003; McDonough, 2006). Nonetheless, the benefits the L2 learners can take from WCF have been doubted by some scholars (Truscott, 1996, 1999, 2007; Truscott & Hsu, 2008). Truscott (1999) rebutted that language teachers could unequivocally expect their error corrections to help L2 learners acquire all linguistic forms and structures, since language learning in its natural and systematic way needs L2 explicit knowledge and a thorough and overall understanding of language form, content, and use. Concerning writing progress, Truscott (2007) believed that since WCF makes little contribution to developing accuracy in L2 writing, it has no place in classroom-based writing. In other words, “improvements made during revision are not the evidence on the effectiveness of correction for improving learners’ writing ability” (Truscott & Hsu, 2008, p. 293).

In the absence of serious examination of WCF long-term efficacy, well-designed and valid experimental studies, the challenge over WCF benefits still persists. Within the WCF advocates party, a group of researchers have been interested in the extent to which different WCF strategies and their amount of explicitness may have divergent impacts on L2 writers’ accuracy (e.g., Bitchener, 2008; Bitchener & Knoch, 2010; Bitchener, Young, & Cameron, 2005; Ferris, 2011). Such choices are by and large dependent on such factors as the types of committed error (i.e., treatable errors such as verb tense or untreatable errors such as word choice), the nature of the writing task (i.e., product- or process-oriented writing), and the students’ proficiency level (Van Beuningen, De Jong, & Kuiken, 2012).

As the controversy grows, only a few frontline writing teachers await plausible research findings or conclusive evidence; instead, they respond to every single error as it occurs based on “their intuition, experience, or students’ needs” (Ferris, 2011, p. 644). Consequently, very little is known or reported about what *actually* happens in L2 writing classroom when teachers respond to errors in students writing. As a matter of fact, ecological studies indicate that L2 writing teachers’ WCF practice is mostly haphazard, and unlikely well-informed by research findings or theoretical principles (Bitchener, 2008; Bitchener & Knoch, 2010).

From a different standpoint, a great need is stressed to address the under-application of WCF research findings in our current situation where large-scale efforts are made to utilize technology in remote learning and distance education during the Coronavirus Disease 2019 (COVID-19) pandemic. Online initiatives, resources and educational programs have emerged and evolved quickly in the absence of face-to-face teacher mediation, wherein the L2 writers can still benefit from WCF. It is through computer-mediated writing assignment or online classroom tasks that the teachers’ suggested notes or corrective feedback can easily be transmitted to the L2 writers. Therefore, it is believed that using modern technological devices such as smart phones, iPads or even desktop computers might create a platform to bring to the L2 learners the advantages of immediate and more favorable types of WCF (Burston, 2014; Kılıçkaya, 2019).

## Background and purpose

Exploring how individuals succeed to acquire L2 reading and writing literacy has always fascinated the SLA theorists and researchers. They seem keenly interested in assisting the L2 learners to overcome the recurrent patterns of errors in the process of L2 writing. The case of under-achieving L2 writers raises the critical question about how errors should be treated; in other words, either should errors be taken as harmful acts to be interrupted or as productive acts to be responded, because of the highlighted awareness they raise about a learner’s level of language acquisition, and the way they can speed up acquiring the explicit knowledge of language forms.

Solid evidence on the necessity to focus on language form was denoted in the French immersion programs in 1974 in Canada, when researchers came to believe that despite the French as a second language (FSL) learners progressed in fluency, communicative abilities, and their confidence in using French, they commonly failed to master French grammar use even after years of extensive exposure (Harley & Swain, 1984). The majority of educators had to unequivocally accentuate the need to respond to the L2 learners’ language production with corrective feedback. The focus-on-form movement (Long, 1991; Long, Inagaki, & Ortega, 1998) which called for the L2 teachers’ immediate but brief focus on students’ errors of language form in the midst of meaning-focused tasks was only one way to achieve such a disregarded goal. L2 teachers were encouraged to respond with detailed and comprehensive feedback to students’ writing with the hope of engaging them with WCF and improving their grammatical accuracy.

Seemingly, a variety of WCF practices have been used to heave up L2 learners’ attention to linguistic structures and to trigger constant self-monitoring of their process of language learning (Hyland, 2002; Storch & Wigglesworth, 2010). Successful awareness-raising techniques in WCF, such as “holding one-on-one writing conferences” (Hyland & Hyland, 2006, p. 90), keeping an “error log” (Ferris & Roberts, 2001, p. 162) or metalinguistic feedback (Hartshorn et al., 2010) are also known “to encourage proactive self-analysis of language learning needs” (p. 45). Since the WCF research has a direct relevance to the work of L2 writing teachers, a systematic approach seems pivotal to examine the reality of WCF in L2 writing context. The literature review in this research paper distinguishes five sets of alternatives in the degree of Exposure to WCF (direct versus indirect), amount of WCF (focused versus unfocused), learnability of WCF (revision versus re-writing the corrected errors), WCF strategies, and error types in WCF (treatable versus untreatable).

### Exposure to WCF

By definition, direct corrective feedback is a practice of “providing some form of explicit written feedback on linguistic form or structures, above or near the linguistic errors” (Ferris, 2003, p. 113). Direct WCF can take the form of crossing out of the unnecessary word(s), inserting missing word(s), and providing the correct form(s) or structure(s) on a written assignment. Direct WCF can also include “metalinguistic feedback (i.e., the provision of grammar rules and examples of correct usage) and sometimes “oral form-focused instructions to clarify the written metalinguistic explanation provided” (Ferris, 2011, p. 294). Indirect WCF, on the other hand, is a kind of notice the teachers give to their students to indicate the occurrence of an error but they intend not to provide any correction henceforth. Indirect WCF is usually provided either by “underlining or circling an error, or by recording in the margin the number of errors in a given line” (Ferris, 2011, p. 297).

Those researchers recommending direct WCF reassure the writing teachers that it is more constructive to L2 writers as it (1) slims the chance of bewilderment that students will usually experience if they fail to recollect the received indirect feedback; (2) grants them a toolkit to predict and prevent future errors; and eventually, (3) seems more intriguing and immediate to the students in writing classrooms (Bitchener & Ferris, 2012). On the other hand, in the absence of teachers’ explicit and direct error correction, the L2 writers are expected to resolve and remove the noted errors by themselves. Therefore, the SLA researchers and L2 teachers advocating indirect WCF believe that this approach is more efficient since it provokes the L2 writers to immerse themselves into self-guided language learning and autonomous problem solving. Hence, it will allow greater cognitive engagement and enhance long-term writing improvement (Ferris, 2004; Hashemian & Farhang-ju, 2018).

As a popular indirect WCF strategy, metalinguistic WCF involves affording language learners some form of coded comments about the essence of the committed errors (Bitchener & Knoch, 2010; Ferris & Roberts, 2001). A number of studies have compared metalinguistic WCF to other strategies and reported contradictory findings. Lalande (1982), for instance, argued that the L2 German learners improved their writing performance after receiving metalinguistic WCF in terms of underling errors, whereas a group who received direct WCF made even more errors in their future writing. Later, however, Robb, Ross, and Shortreed (1986) who implemented a systematic error coding feedback in their research, reported the metalinguistic WCF as less practical to both teachers and students than other WCF strategies (i.e., direct, electronic, and reformulation feedback). Ferris (2003) also conducted a longitudinal comparative study about the impact of metalinguistic WCF on the frequency of errors in her students’1st and 4th essays in a writing course and her findings showed “improvement in total error numbers and verb errors but not in noun errors, article errors, lexical errors, or sentence errors” (p. 230). Overall, there is no conclusive evidence to imply that metalinguistic WCF can consistently benefit the L2 writers to achieve writing accuracy over time; hence, further experimental research seems inevitable.

### Amount of WCF

Processing and acquiring all corrected errors are challenging for most L2 learners after receiving comprehensive (i.e., unfocused) WCF if they have to attend to all committed errors at the same time. While to several researchers, unfocused WCF is detailed and authentic; many more blamed it as an unsystematic WCF strategy which overloads L2 writers’ working memory capacity (Ellis et al., 2006). The writing teachers, therefore, are encouraged to select a single or a few rule-governed error categories for giving feedback, such as articles, verb tense or subject-verb agreement. On the other hand, the unfocused WCF is favored for its benefit of “addressing a wide range of errors, so while it might not be as effective in assisting learners to acquire specific features as focused WCF in short term, it may prove superior in the long run” (Gholami & Narimani, 2012, p. 51).

Focused or selective WCF seems more manageable and practical as the learners can examine several corrections of only one error type and receive abundant evidence they need to both notice and understand their errors (Bitchener & Knoch, 2010). In other words, “if learning is dependent on attention to form, then it is reasonable to assume that the more intensive the attention, the more likely the correction is to lead to learning” (p. 42). In the same vein, focused metalinguistic WCF may benefit the L2 learners in many ways as it endorses both L2 learners’ attention and realization of the *nature* of their errors (Sheen, 2007). The majority of studies in L2 context have examined only the unfocused type of WCF (e.g., Ferris & Roberts, 2001; Hashemian & Farhang-ju, 2018; Semke, 1984) which impress the researchers to counter-examine the efficacy of these two WCF strategies in classroom writing practice.

### Learnability of WCF

A critical issue in examining the impacts of WCF is *how* L2 learners respond to the received feedback. In a process-writing classroom, L2 learners’ uptake is expected in terms of the error-free revision of the initial draft (e.g., Hashemian & Farhang-ju, 2018; Van Beuningen et al., 2012). However, in the majority of writing tasks in the product-oriented out-of-class or online contexts, it is compulsory for the students to simply pick out the corrected errors and re-write them, irrespective to the students’ constructive engagement with the feedback (e.g., Ferris & Roberts, 2001).

Several research findings suggested that WCF is instrumental and productive only if it is responded to the interim rather than terminal drafts (Bitchener, 2008; Ferris, 2011). In a renowned experiment, for instance, Ferris (2011) collected 146 ESL students’ revised compositions and found that after receiving WCF, more than 80% of the errors were eliminated in the revised essays by “(1) correcting the error; (2) deleting the text containing the error, or (3) making a correct substitution” (p. 649). The study strongly supported the role of WCF as an intervention strategy which encourages the L2 writers to notice the gap between what they can produce and what they aim to produce. In terms of language acquisition, however, such a conclusion seems marginal, so as Truscott and Hsu (2008) argued, simply reporting that WCF can assist students to correct errors and revise their writing has no solid implication in whether they have *acquired* the corrected errors or whether they can control them notably in future as a long-term effect. In other words, from the SLA perspective, small or short-term changes can hardly be the evidence of acquisition (Bitchener & Knoch, 2010). The concerning issue of L2 writers’ long-term uptake is so pivotal that it often restricts the teachers’ conventional choice of WCF strategies in favor of alternative forms of WCF such as peer feedback, self-editing or mobile and computer-assisted WCF (Van Beuningen et al., 2012).

### Alternative WCF strategies

In recent years, mobile-assisted language learning (MALL) has made entrance to most educational settings, especially L2 learning/teaching context. So far, the power of wireless technologies has improved markedly, “especially with smart mobile phones which have incorporated the functionality of hand-held computers and audio-video recorders and players” (Jalilifar & Mashhadi, 2014, p. 110). Among the pedagogical merits of mobile language learning are its adaptation to the students’ learning styles and preferences (Dashtestani, 2015), versatile multimedia capabilities (Kukulska-Hulme & Shield, 2008), “ubiquitous Internet connectivity, interactive and dynamic learning environment” (Jalilifar & Mashhadi, 2014, p. 112), increasing “students’ motivation, cost-effectiveness, and student-friendliness” (Milrad & Jackson, 2008, p. 86), and growing teacher-student communication (Stockwell, 2010; Walker, 2013). Such countless benefits have provoked a number researchers and educators to see MALL as an abiding type of learning facility and to shift their attention from *e*-learning to *m*-learning (Petersen & Divitini, 2005). Likewise, several studies have surveyed the L2 learners’ attitudes towards mobile-mediated WCF, and reported their indisputable support for m-learning as an invaluable instructional medium for improving writing skills (Arnold & Ducate, 2011; Burston, 2014; Dashtestani, 2015; Ho & Savignon, 2007; Jalilifar & Mashhadi, 2014; Yeha & Lob, 2009). They also studied the viable effects of WCF in MALL environment on teacher-student and student-student interactions (Mohammadi, Jabbari, & Fazilatfar, 2018). Yet, there are a few empirical studies (Anaraki, 2009; Begum, 2011) that have closely examined the virtual L2 teachers’ WCF practice responding to the L2 writers in the mobile-mediated contexts.

### Error types in WCF

Although there has been plenty of research (e.g., Ferris, 2003; Ferris & Roberts, 2001; Hashemian & Farhang-ju, 2018; Pearson, 2020) which supported the role of WCF in improving L2 writers’ control over rule-based, treatable grammatical forms and structures, the extent to which various WCF strategies might be helpful in treating other non-rule-governed (e.g., lexical choices, word order) and more complex (e.g., discourse markers) linguistic forms have remained unknown and demand further research. On the other hand, a long-term preoccupation with language forms has made many L2 writing teachers ignore the multifaceted nature of writing competence and the fact that the real ability to write requires the L2 learners to control the content, to practice creativity and to use language for communication at discourse level. As Hedge (1988) restated, among the critical factors that make writing even more effective are the well-established organization of ideas, high accuracy with complex discourse devices, besides the proper use of vocabulary and a suitable style according to subject matter and prospective readers. In other words, although mastery of vocabulary and grammar is unquestionable strength in L2 writers, what makes a piece of writing more appealing is having cohesion at lexico-grammatical and coherence at pragmatic levels which cannot be accomplished through rule-governed grammatical structures per se (Dergisi, 2010; Mohammadi et al., 2018).

By definition, discourse markers are “linguistic elements which signal relations between units of talk by virtue of their syntactic and semantic properties and by virtue of their sequential relations as initial or terminal brackets demarcating discourse units” (Schiffrin, 1987, p. 40). Halliday and Hasan (1976) stated that discourse markers are critical means of generating coherence in a meaningful stretch of spoken or written discourse. Conjunctions, which Halliday and Hasan (1976) termed as discourse markers (DMs) are defined as connective elements, which glue different parts of a text at various levels of clauses, sentences, and paragraphs. They divided DMs into four types of additive, adversative, causal and temporal devices. On one hand, recent studies in L2 pedagogy have demonstrated the considerable impacts of instructions to using DMs on L2 learners’ accuracy of spoken and written performance (Alraddadi, 2016; Assadi Aidinlou & Shahrokhi Mehr, 2012; Daif-Allah & Albesher, 2013, to name a few). On the other hand, such accuracy in using DMs has been promoted through either teaching them directly/explicitly or raising L2 learners’ awareness by analyzing their committed DM errors in their oral or written discourse (Hamed, 2014; Vickov & Jakupcevic, 2017). In either case, the experience of learning DMs is commonly generated in actual classroom setting, where the L2 teacher and learners are involved in face-to-face interaction, with a few such attempts in computer-assisted or mobile-mediated L2 learning virtual environment.

## Research design

The current study benefited from both qualitative and quantitative data which were collected and analyzed concurrently to examine the raised research questions. Adopting the convergent mixed-methods design (Creswell & Creswell, 2018), the researchers in this study investigated.

1. To *what extent* metalinguistic WCF affects EFL learners’ accuracy of four types of DMs in their writing performance, and.
2. *How* metalinguistic WCF affects EFL learners’ accuracy of DMs in their writing performance over an eight-week period.

The metalinguistic WCF exclusively addressed four types of discourse markers; that is, additive, adversative, causal and temporal DMs (Halliday & Hasan, 1976) typology, as by several research reports, they have been reported as necessary devices to elevate coherence and comprehensibility in writing performance (Alraddadi, 2016; Jalilifar, Shooshtari, & Mutaqid, 2011). Meantime, the metalinguistic WCF was given and received only through the mobile application of *WhatsApp* messenger as the pedagogical mobile-mediated environment.

## Method

### Participants

An initial intact group of 45 Iranian EFL learners (25 female and 20 male) consented to voluntary participation among whom three volunteers were excluded after administering the Oxford Preliminary Test (2004 version) as the placement test in this study. Within the age range of 19 to 45 and average of 28, the 42 candidates enrolled in a virtual course of English paragraph writing at a private language institute in Karaj, Iran which lasted for eight consecutive weeks. The participants were undergraduate university students majoring in engineering (27%), medical sciences (22%), accounting (22%), Russian literature (16%) and architecture (13%), whose general English proficiency was determined at advanced levels of C1 and C2, trusting their range of obtained scores (48–60) on the OPT.

### Instruments

#### Oxford Preliminary Test (OPT) (Allan, 2004)

OPT is a standard test of general English proficiency with a six-point rating scale. As stated in www.oxfordenglishtesting.com (2020), OPT “measures a test taker’s ability to understand a range of grammatical forms and the meanings they convey in a wide range of contexts” (p. 1). The candidates whose scores fall between 0 and 17 are assigned to basic (A1) level, 18–29 to elementary (A2), 30–39 to intermediate (B1), and 40–47 to upper-intermediate (B2) levels. To the advanced ranking positions, the test takers whose scores are between 48 and 54 (C1), and 55–60 (C2) are assigned.

At the outset of the experiment, to minimize the odds of initial differences among the participants in terms general English proficiency, the researchers administered a 2004 version of OPT with 100 multiple-choice items and reasonable measure of inter-item reliability (Cronbach *α* = 0.883). Eventually, 42 students whose OPT scores assigned them to levels of C1 and C2 were selected as the final research sample.

#### WhatsApp Messenger

In this study, the metalinguistic WCF was mediated through the mobile-assisted messaging application of WhatsApp due to its suitability and a rather wide range of applicability in Iran. The WhatsApp Messenger is a free software which works both on mobile devices and desktop computers. This freeware service needs its users to have access to the Internet and own a standard mobile number for registration. The users can send “text and voice messages, make voice and video calls, and share images and photos, documents and other media” via WhatsApp Messenger (Metz, 2016, p. 13).

In this study, WhatsApp Messenger created a mobile-mediated L2 learning platform for sending and receiving metalinguistic WCF. The participants’ access to the instructor, who was one of the researchers in this study, was only possible by sending their scripts to the instructor’s private message and receiving the instructor’s WCF addressed to their written scripts in the WhatsApp public chatroom.

#### Writing elicitation tasks

Eight writing elicitation tasks were introduced to the participants in terms of provoking writing topics illustrated with some pictorial cues. The students were required to write a 150–200 word paragraph about every topic in a weekly schedule for an 8 week period (Table 1).

**Table 1:** Writing Elicitation Tasks

Session	Topic
1	- Imagine living 1 day with no plants or trees on the earth. How different would your life and your town look like?
2	- Think of a day when a robot brings you tea or helps you cleaning your room. What do you think of it? Good or bad?
3	- Have you experienced unfair judgments in your life? When and how?
4	- Imagine a day without the Internet, smart phones or computers. How would your life most likely become?
5	- Do you agree with this saying that “TV prevents us from thinking”?
6	- What do you think about boys finding jobs like being a pilot or a soldier, and girls some jobs, like child nursing or teaching at school?
7	- Do you see university degrees as necessary for you future employment?
8	- Why do most people still prefer living in cities rather than country sides?

## Data collection procedure

Three days before the treatment began, the participants were provided with a brief tutorial on the research plan and an opportunity to ask their questions from the researchers. Data collection procedure was conducted in the following steps:

1. On day one, the OPT was administered as the placement test to screen and eliminate the participants (*n* = 3) whose scores were below the advanced level of language proficiency (C1 and C2, in OPT rating scale). The final 42 selected candidates were informed that in the following 8 weeks they were required to write a 150–200 word paragraph on every selected topic and send it to the instructor through WhatsApp Messenger. Next, they received detailed instructions on four types of DMs (Halliday & Hasan, 1976) through exemplar sentences which lasted for 2 h. After reassuring of the participants’ comprehension of DMs with some essay-type written questions, the researchers distributed a glossary of the types of DMs with selected examples among the participants.
2. Two days later, the participants were asked to write a 150–200 word paragraph on the first assigned topic and to send it to the instructor’s private massage within 24 h.
3. Within 6 h after the participants sent their assignment, it was endorsed with metalinguistic WCF and uploaded by the instructor on the WhatsApp public chatroom to be observed and reviewed by all participants. The current researchers collaborated on providing metalinguistic feedback.
4. Once a DM error was located by the researchers, it was highlighted and an abbreviation above the error (e.g., *ADD* for additive DM, or *AD* for adversative DM) referred the students to the following explanations in the DMs glossary sheet:
  ADD = Additive DM, such as *and, also, moreover, in other words,* is used to repeat or emphasize the previous information or add relevant new ideas or expressions to them.
  AD = Adversative DM, such as *yet, though, however, in any case, instead,* is used to express contrasts and comparisons between sentences or corrections in relation to foregoing discourse.
  CD = Causal DM, such as *so, then, therefore*, *because,* is used to signal the results or consequences of what is being stated*.*
  TD = Temporal DM, such as *next, when, secondly,* is used to reflect the chronological, logical or sequential relationship between discourse units. No further error categories were corrected or received coded feedback.
5. The participants were required to correct their errors, revise the initial drafts according to the coded feedback, and try to eliminate them in their new drafts in the following weeks

## Results

### Analysis of qualitative data

The thematic analysis of the participants’ written scripts followed the operationalizing DMs in Halliday and Hasan’s (1976) typology. Responding to errors and providing metalinguistic WCF were carried out by all researchers in this study. The content of collected writings was inserted into SQR NVivo 10 software and the distribution of extracted themes for four types of DM was retrieved. To further elaborate on the distribution of DMs, the written scripts of a random participant (AR) was collected in Sessions 1 (first session), 4 (midsession), and 8 (last session) of the intervention. Table 2 shows the AR’s writing on the topic of Session 1, *Imagine living 1 day with no plants or trees on the earth. How different would your life look like?*, as well as the frequency of the received coded feedback and the thematic distribution of the DMs.

**Table 2:** Session 1: Data extract with frequency of coded feedback and themes for AR

Example data extract	Coded Feedback	f	Themes	f
I have to say that there is no world without plants. That’s for us anyway. One might think the most important function of trees *and* plants is to produce oxygen. That is not the case. *However*, the Oxygen provided by plants is vital to insurance of our lives that is only one part of a very complicated equation. What plants do underneath the ground is extremely important. And their function is creating water canals. Plants *and* trees have roots underground. These roots join *and* create a web that enforces soil structure *and* holds the soil together like glue. Without them water runs free *and* spreads on the ground to be evaporated fast *before* absorption by the ground. Roots guide the water flow *and so* the water goes deeper *and so* streams appear *and* those turn into rivers that are the very foundations of our civilizations. *When* the plants *and* trees are gone, the earth will change drastically. The form of the soil changes *and* soon come wild sandstorms.	ADD	1	Additive	10
			Adversative	1
			Causal	1
			Temporal	2

As Table 2 shows, in Session 1, AR used all types of DMs (*n* = 15), including *and, however*, *before*, *so*, *if*, 10 of which were additive DM. Among additive DM, *and* was overused. Table 3 displays the frequency of AR’s received coded feedback and the thematic distribution of the DMs, in Session 4, on the topic of *Imagine a day without the Internet, smart phones or computers. How would your life most likely become?*

**Table 3:** Session 4: Data extract with frequency of coded feedback and themes for AR

Example data extract	Coded Feedback	f	Themes	f
To be honest, it is really difficult to imagine. I believe that would cause great difficulty for businesses *and* big companies *because* they rely heavily on technology to enhance their production rate, services and the employees, *and* employers use these instruments to communicate. Ordinary people depend on technology as well*. Initially*, I thought that it means no more noisy Turkish series, *but then* I remembered about banking transactions *and* bill payment that we conveniently use our smartphones for. Internet is the key of all the uses of smartphones *as well as* computers, *since* it connects all the devices to the servers *and* enables governments, scientists, industries, *and* etc. to do various things. *Therefore,* even if we had the devices, without the internet services, we wouldn’t be able to utilize them properly. *Moreover,* I think we need to appreciate the opportunities that these tools offer us *when* sometimes we ignore how amazing they are.	TD	1	Additive	7
			Adversative	2
			Causal	2
			Temporal	3

In Table 3, it can be seen that all four types of DMs (*n* = 14) were used more reasonably and frequently. Moreover, AR started to use less frequent DMs, such as *initially, since,* and *as well as* which show some degree of adjustment in her writing performance. Still, AR used more additive DM (*n* = 7) than other three DM types. Finally, as Table 4 displays the essay written in Session 8, AR wrote on the topic of *Why do most people still want to live in cities rather than country sides?*

**Table 4:** Session 8: Data extract with frequency of coded feedback and themes for AR

Example data extract	Coded Feedback	f	Themes	f
*Although* there are many problems in the cities, people still want to live in them *because* of one simple reason: Civilization. Civilization is the core of our success as a species. It has provided us with safety *and* comfort that allows steady progress *and* development. *Too,* the cities provide a bed of opportunities giving people chances to do things that they can’t do in small scales. There is more work in a city *so* there’s also more money as well. The very population of the city is its advantage *as* it gives people security from outside world. Life in villages *and* less developed cities might be more difficult specially, if you live in third world countries caused by the lack of attention *and* less efficient utilities in such areas. Rural life is often easier in first world countries *as* they provide methods to live in more comfort, *but* even that doesn’t stop people from migrating to big cities.	ADD	1	Additive	6
			Adversative	2
			Causal	4
			Temporal	0

According to Table 4, in Session 8, despite the absence of the temporal DM in AR’s writing (*n* = 0), the instances of other types of DMs, such as *although, because of, and, also,* and *so* were accomodated accurately with similar frequency rates (*n* = 14). To sum up, the frequency distribution of four types of DMs in AR’s writing was different in every session, with no sign of considerable improvement over 8 weeks of receiving metalinguistic WCF. Similar unsystematic and fluctuating patterns of DMs distribution were retrieved and reported in other participants’ writing.

### Analysis of quantitative data

#### Exploring first research question

Following Ellis and Barkhuizen (2005), accuracy on every obligatory occasion of DMs usage was calculated as “a percentage of correct usage for all occasions where the grammatical structure of the sentence written by the students required it” (cf. Ellis & Barkhuizen, 2005, p.73, for a thorough discussion of *obligatory occasion analysis*). For instance, in a written script, the three correct usage of the additive DM from 10 obligatory occasions was calculated as 30% accuracy rate.

For frequency analysis of the collected quantitative data, the IBM SPSS 21 descriptive statistics was deployed. The obligatory occasions for the DMs were identified and noted in all the written scripts by each candidate on the eight assigned writing tasks. The interrater reliability was checked across the current researchers’ ratings to ensure the credibility of the content analysis and it had the index of 0.815 in terms of Cohen’s kappa coefficient. The measures of mean and standard deviation for four types of DMs were summarized in Table 5.

**Table 5:** Descriptive Statistics for DMs Distribution in Eight Sessions of WCF

Session	Additive		Adversative		Causal		Temporal	
	M	SD	M	SD	M	SD	M	SD
1	58.00	3.05	12.00	3.00	13.06	1.00	10.00	3.00
2	46.00	3.00	11.00	5.00	15.00	5.00	16.00	2.00
3	47.00	5.00	14.00	6.00	17.00	4.00	10.00	2.00
4	56.00	3.00	11.06	5.00	15.00	5.00	12.00	3.00
5	54.00	6.00	15.00	3.00	13.00	1.00	11.00	1.00
6	60.00	4.00	14.00	3.00	16.00	3.00	15.00	1.00
7	50.00	3.00	14.00	5.00	11.00	4.00	10.00	3.00
8	58.00	4.00	15.00	3.00	18.00	4.00	10.05	3.00

**Fig. 1:**
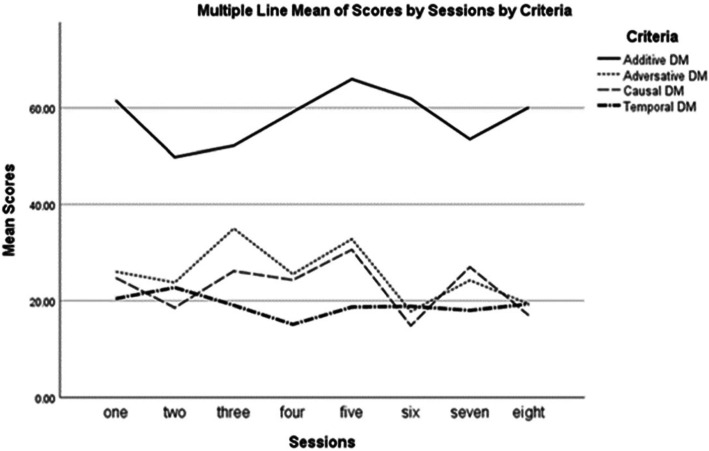
Frequency distribution by the mean scores of four types of DMs in eight treatment sessions

As it can be seen in Table 5, the measures of mean for additive DM are much larger than the other three types of DMs in all eight writing tasks, ranging from M = 46 in Session 2 to M = 60 in Session 6. To explore the impact of metalinguistic WCF on each type of DMs accuracy raised in the first research question on *the extent* to which metalinguistic WCF affects EFL learners’ accuracy of four types of DMs in their writing performance, the measures of mean and SD demonstrated fluctuating patterns of distribution across four types of DMs, despite the participants constantly using additive DM more than the three other DM types along eight sessions of metalinguistic WCF. In other words, through the treatment sessions, no considerable progress in the DMs accuracy could be seen in participants’ writing after receiving metalinguistic WCF. Figure 1 provides a visual representation of the mean distributions for four types of DMs.

As Fig. 1 illustrates, the additive DM was consistently used more frequently than the other three types of DM since the first session of the treatment to the end; however, the frequency patterns of all DMs seemed constant with no considerable progress over the sessions of metalinguistic WCF.

#### Exploring second research question

To explore the second research question which inquired *how* metalinguistic WCF would affect EFL learners’ accuracy of DMs in their writing performance over an eight-week period, or in another terms, the significance of fluctuations in DMs accuracy along the metalinguistic WCF intervention sessions, the frequency pattern for every type of DMs was cross-examined in a contingency table (Table 6).

**Table 6:** Contingency Table for Metalinguistic WCF Duration and DMs Accuracy

Session		Additive	Adversative	Causal	Temporal	Total
1	Count	548	240	199	173	1160
	% within Session	58.9%	12.7%	18.1%	10.3%	100.0%
	Adjusted Residual	3.5	−5.1	2.0	−1.5	
2	Count	507	237	172	184	1100
	% within Session	46.1%	21.5%	15.6%	16.7%	100.0%
	Adjusted Residual	−5.6	3.0	−.4	5.6	
3	Count	521	271	170	178	1140
	% within Session	47.4%	24.6%	15.5%	12.5%	100.0%
	Adjusted Residual	−4.7	5.8	−.6	1.0	
4	Count	619	232	185	164	1200
	% within Session	56.3%	21.1%	16.8%	5.8%	100.0%
	Adjusted Residual	1.6	2.6	.7	−6.4	
5	Count	597	186	207	181	1171
	% within Session	54.2%	16.9%	18.8%	10.1%	100.0%
	Adjusted Residual	−.1	−1.3	2.7	−1.7	
6	Count	665	156	197	172	1190
	% within Session	60.5%	14.2%	12.5%	12.9%	100.0%
	Adjusted Residual	4.6	−3.8	−3.5	1.4	
7	Count	554	220	203	173	1150
	% within Session	50.4%	20.0%	18.5%	11.2%	100.0%
	Adjusted Residual	−2.6	1.6	2.3	−.5	
8	Count	640	167	138	168	1113
	% within Session	58.6%	15.3%	12.6%	13.5%	100.0%
	Adjusted Residual	3.2	−2.8	−3.3	2.1	
Total	Count	4651	1709	1471	1393	9224
	% within Session	54.0%	18.3%	16.0%	11.6%	100.0%

Table 6 shows the patterns of distribution for all types of DMs over the eight-week period of treatment, along which additive DM sustained its outnumbered appearance from the first to the last sessions. While the distribution of additive DM fluctuates within the largest continuum of 507 (Session 2) to 665 (Session 6) occurrences, the temporal DM falls down slowly from 184 (Session 2) to 164 (Session 4) occurrences. In the middle ground, the distribution of two types of adversative DM within the range of 156 (Session 6) to 271 (Session 3), and casual DM within the range of 138 (Session 8) to 207 (Session 5) occurrences appeared. Meanwhile, the total number of DMs was 9224 with a rather small range of fluctuation (1100–1200) in the eight-week treatment period. To investigate the significance of the observed within-group fluctuation across four types of DMs, an 8 × 4 group-independence Chi-square was calculated between the duration of metalinguistic WCF and DMs accuracy of use.

Summarized in Table 7, the measure of Pearson Chi-square was statistically significant [*χ*^*2*^ = 221.548_(21, 8794)_, *p* = 0.000, Cramer’s V = 0.910 (indicating a strong effect size)]. To sum up the findings, the frequency counts of the four types of DMs showed different distributions in the participants’ writing, with the additive DM as the most frequent (*n* = 4651) and temporal DM as the least (*n* = 1393) overall. However, such fluctuating patterns of occurrence seemed consistent and non-significant along the eight-week period of treatment rendering no accountable sign of improvement in participants’ writing performance through and after receiving metalinguistic WCF.

**Table 7:** Chi-Square Test: The DMs Accuracy by the Metalinguistic WCF Duration

	Value	df	Asymptotic Significance (2-sided)
Pearson Chi-Square	211.548	41	.000
Likelihood Ratio	217.612	41	.000
Linear-by-Linear Association	4.249	1	.039
Cramer’s V	0.910		.000
N of Valid Cases	9224		

## Discussion

This study aimed at investigating the extent and the quality of metalinguistic WCF possible impacts, specifically focused on EFL learners’ use of discourse markers in a mobile-mediated language learning platform. Qualitative data analysis in this study indicated a considerable divergence in the frequency patterns of four types of DMs (i.e., additive, adversative, causal, temporal) in the participants’ writing performance. To be precise, the additive DM had an outnumbered appearance over adversative, causal and temporal DMs. Much to the chagrin of the current researchers, however, despite a significant difference in the frequency distributions of the DMs, the statistical data analysis failed to support a meaningful improvement in the participants’ DMs accuracy along an eight-week period of receiving metalinguistic WCF. There is a growing body of research which still examines such nuances and niches in applying WCF. Despite ample empirical evidence, it is still unclear whether specific error types are more sensitive to WCF than others (Bitchener, 2008; Bitchener & Ferris, 2012; Ellis et al., 2006). In line with Truscott’s counterargument (Truscott, 1996; 1999; 2007) that WCF should be abandoned as it is ineffective or even harmful to the L2 learners, this study also came up with findings which disputed several researchers who have supported WCF to have a constructive role in improving L2 writing accuracy (e.g., Bitchener et al., 2005; Ferris & Helt, 2000; Hashemian & Farhang-ju, 2018; Long et al., 1998; Rahimi Domakani, Roohani, & Abdollahian, 2010; Rassaei, 2017; Rassaei, Moeinzadeh, & Youhannaee, 2012).

Conforming to Truscott’s (2007) stark dispute over the effectiveness of WCF, the current researchers posed two main reasons behind their controversial findings. First and foremost, WCF practice might unequally address different linguistic categories (e.g., lexical, grammatical, and discursive forms), so that those language forms which are acquired through seemingly different cognitive and linguistic processes might react unpredictably and equivocally to WCF with no indicative record of L2 writers’ progress. In the same line of arguments, Ferris (2003) made a distinction between treatable (rule-governed) and untreatable errors and claimed that in order to be effective, WCF should be only directed to treatable errors, such as articles, verb-subject agreement and verb tense. In this study, however, the researchers’ choice of error type was the participants’ use of discourse markers which can be labeled as untreatable errors in Ferris’ labeling system (Ferris, 2003). Secondly, as also empirically verified by Meisel, Clahsen, and Pienemann (1981), various WCF strategies (including metalinguistic WCF) are commonly assumed as imposed negative evidence that may improve L2 writers’ performance *temporarily* but they hardly advance their internal grammar which is acquired and internalized over time and only through natural and extensive exposure to L2. In other words, regarding the constructive role of feedback, positive evidence is a trigger for progressive change; negative evidence seems irrelevant in a long shot.

## Conclusion and implications

There have been abiding debates between those who strongly support the advantages of WCF to natural improvement of L2 learners’ writing performance and those who are against them. Likewise, Guénette (2007) argued that research findings on WCF have been so controversial that L2 teachers who try their instructional options of *to correct* or *not to correct* their students’ written performance are left in the middle of contention.

Scholars like Truscott and Hsu (2008), who believed that corrective feedback is futile, insisted that it would result in “embarrassment, anger, inhibition, and feelings of inferiority” in language learners (p. 441). Ellis et al. (2006), Hillocks Jr. (1986) and Knoblauch and Brannon (1981) who reviewed and analyzed several L1 studies also reported that students who had no experience of error correction showed more affirmative attitude and constant resilience toward writing than those who did. In the absence of WCF, the students may not become better writers, yet they intend to write more, perhaps because of their better attitude. Therefore, L2 writing teachers should not undermine the value of positive attitude in itself.

In L2 research domain, several studies found corrective feedback even harmful rather than simply ineffective (Farrokhi et al., 2017; Gu & Wang, 2008; Truscott, 2007). The probable cause of such unforeseen outcome is perhaps the ugliness and pain of error correction. The disrupted L2 writers would probably shorten their writing in order to avoid upcoming error correction; hence, they do not progress in their writing ability as long as they have been developing a calculating, cautious attitude toward language learning.

Although attempts were made to minimize the probable lapses in research design, data collection and content analysis in this study, there are some limitations and implications which should be pointed out once further research is designed in the future. The focus of metalinguistic WCF in this study was only on locating and responding to discourse markers, which can be both a source of strength and a cause of restraint to the generalizability of the findings. Adopting DM lenses made it possible to focus on the EFL learners’ accuracy of cohesive devices; however, further research is needed to estimate the extent to which the findings in this study are applicable to other non-treatable error categories as well.

Further research is also needed to investigate whether alternative means of data collection, such as writing tasks of different genres or various strategies of WCF might change the algorithm of DMs occurrences in EFL learners’ writing. It should also be noted that the participants in this study were Iranian EFL learners at the advanced level of English language proficiency who studied English in the formal educational setting and had received some earlier instructions to the use of DMs in academic English writing and grammar courses. Further research seems necessary to determine whether a variety of WCF strategies would help the elementary or less proficient EFL learners to develop accuracy in using DMs in their writing performance.

Finally, as several incompatibilities were earlier reported between the WCF empirical research findings and real classroom practice (e.g., Hobbs, 2001), the need for more ecological studies in classroom-based contexts to explore the applicability of various WCF strategies, their drawbacks and benefits seems urgent and consequential. This necessity accompanied with the global rise of COVID-19 calls for the adoption of more online education and L2 learning in particular. Therefore, further research on WCF seems inevitable to elevate the online platforms in order to provide the L2 writers with remote or online instructions and the writing teachers with virtual WCF strategy training courses.

## Data Availability

Please contact the authors for data requests.

## Abbreviations

AD: Adversative discourse marker
ADD: Additive discourse marker
CD: Causal discourse marker
COVID-19: Coronavirus Disease 2019
DM: Discourse marker
EFL: English as a Foreign Language
L2: Second/Foreign language
OPT: Oxford Preliminary Test
SLA: Second Language Acquisition
SPSS: Statistical Package for Social Sciences
TD: Temporal discourse marker
WCF: Written corrective feedback

## References

[CR1] Allan D (2004). Oxford placement test 2.

[CR2] Alraddadi BM (2016). The effect of structural discourse markers in an EFL classroom setting. English Language Teaching.

[CR3] Anaraki, F. (2009). A flash-based mobile learning system for learning English as a second language. In *Proceedings International Conference on Computer Engineering and Technology*, (pp. 400–404). Singapore.

[CR4] Arnold N, Ducate L (2011). Present and future promises of CALL: From theory and research to new directions in language teaching.

[CR5] Assadi Aidinlou N, Shahrokhi Mehr H (2012). The effect of discourse markers instruction on EFL learners’ writing. World Journal of Education.

[CR6] Begum R (2011). Prospect for cell phones as instructional tools in the EFL classroom: A case study of Jahangir Nagar University, Bangladesh. English Language Teaching.

[CR7] Bitchener J (2008). Evidence in support of written corrective feedback. Journal of Second Language Writing.

[CR8] Bitchener J, Ferris DR (2012). Written corrective feedback in second language acquisition and writing.

[CR9] Bitchener J, Knoch U (2010). The contribution of written corrective feedback to language development: A ten month investigation. Applied Linguistics.

[CR10] Bitchener J, Young S, Cameron D (2005). The effect of different types of corrective feedback on ESL student writing. Journal of Second Language Writing.

[CR11] Burston J (2014). MALL: The pedagogical challenges. Computer Assisted Language Learning.

[CR12] Creswell WJ, Creswell JD (2018). Research design: Qualitative, quantitative, and mixed methods approaches.

[CR13] Daif-Allah AS, Albesher K (2013). The use of discourse markers in paragraph writings: The case of preparatory year program students in Qassim University. English Language Teaching.

[CR14] Dashtestani, R. (2015). Moving bravely towards mobile learning: Iranian students’ use of mobile devices for learning English as a foreign language. *Computer-Assisted Language Learning*. 10.1080/09588221.2015.1069360.

[CR15] Dergisi USA (2010). Discourse markers in English writing. The Journal of International Social Research.

[CR16] Ellis R, Barkhuizen G (2005). Analyzing learner language.

[CR17] Ellis R, Loewen S, Erlam R (2006). Implicit and explicit corrective feedback and the acquisition of L2 grammar. Studies in Second Language Acquisition.

[CR18] Farrokhi F, Zohrabi M, Chehr Azad MH (2017). The effect of the corrective feedback on Iranian EFL learners' speaking accuracy and breakdown fluency. Journal of Language Horizons.

[CR19] Ferris DR (2003). Response to student writing: Implications for second language students.

[CR20] Ferris DR (2004). The “grammar correction” debate in L2 writing: Where are we, and where do we go from here? (and what do we do in the meantime . . .?). Journal of Second Language Writing.

[CR21] Ferris DR, Hyland K, Hyland F (2006). Does error feedback help student writers? New evidence on theshort- and long-term effects of written error correction. Feedback in second language writing: Contexts and issues.

[CR22] Ferris DR, Hinkle E (2011). Written discourse analysis and L2 teaching. Handbook of research in second language teaching and learning.

[CR23] Ferris DR, Helt M (2000). Was Truscott right? New evidence on the effects of error correction in L2 writing classes.

[CR24] Ferris DR, Roberts BJ (2001). Error feedback in L2 writing classes: How explicit does it need to be?. Journal of Second Language Writing.

[CR25] Gholami J, Narimani E (2012). Consciousness-raising through written corrective feedback: The case of marked third person -s. Journal of Research in Applied Linguistics.

[CR26] Gu S, Wang T (2008). The impact of negative feedback, noticing, and modified output on EFL question development. Foreign Language Teaching and Research.

[CR27] Guénette D (2007). Is feedback pedagogically correct?: Research design issues in studies of feedback on writing. Journal of Second Language Writing.

[CR28] Halliday MAK, Hasan R (1976). Cohesion in English.

[CR29] Hamed M (2014). Conjunctions in argumentative writing of Libyan tertiary students. English Language Teaching.

[CR30] Harley B, Swain M, Davies A, Criper C, Howatt APR (1984). The interlanguage of immersion and its implications for second language teaching. Interlanguage.

[CR31] Hartshorn JK, Evans NW, Merrill PF, Sudweeks RR, Strong-Krause D, Anderson NJ (2010). The effects of dynamic corrective feedback on ESL writing accuracy. TESOL Quarterly.

[CR32] Hashemian M, Farhang-ju M (2018). Effects of metalinguistic feedback on grammatical accuracy of Iranian field (in) dependent L2 learners’ writing ability. Journal of Research in Applied Linguistics.

[CR33] Hedge T (1988). Writing.

[CR34] Hillocks G (1986). Research on written composition: New directions for teaching.

[CR35] Hino J (2006). Linguistic information supplied by negative feedback: A study of its contribution to the process of second language acquisition (Unpublished doctoral dissertation).

[CR36] Ho MC, Savignon SL (2007). Face-to-face and computer-mediated peer review in EFL writing. CALICO Journal.

[CR37] Hobbs G (2001). Academic journal publishing: Past, present and future. Journal of Education for Teaching.

[CR38] Hyland K (2002). Teaching and researching writing.

[CR39] Hyland K, Hyland F (2006). Feedback on second language students’ writing. Language Teaching.

[CR40] Iwashita N (2003). Negative feedback and positive evidence in task-based interaction: Differential effects on L2 development. Studies in Second Language Acquisition.

[CR41] Jalilifar A, Mashhadi A (2014). Current trends in research on mobile phones in language learning. Research in Applied Linguistics.

[CR42] Jalilifar A, Shooshtari Z, Mutaqid S (2011). The effect of hedging instruction on reading comprehension for Iranian University students. Research in Applied Linguistics.

[CR43] Kılıçkaya, F. (2019). Pre-service language teachers’ online written corrective feedback preferences and timing of feedback in computer-supported L2 grammar instruction. *Computer-Assisted Language Learning*, 1–25. 10.1080/09588221.2019.1668811.

[CR44] Knoblauch CH, Brannon L (1981). Teacher commentary on student writing: The state of the art. Freshman English News.

[CR45] Kukulska-Hulme A, Shield L (2008). An overview of mobile assisted language learning: From content delivery to supported collaboration and interaction. ReCALL.

[CR46] Lalande JF (1982). Reducing composition errors: An experiment. Modern Language Journal.

[CR47] Li, S. (2010). The Effectiveness of Corrective Feedback in SLA: A Meta‐Analysis. *Language Learning, 60*(2), 302-365

[CR48] Long M, Inagaki S, Ortega L (1998). The role of implicit negative feedback in SLA: Models and recasts in Japanese and Spanish. Modern Language Journal.

[CR49] Long MH, De Bot K, Ginsberg RB, Kramsch C (1991). Focus on form: A design feature in language teaching methodology. Foreign language research in cross-cultural perspective.

[CR50] McDonough K (2006). Interaction and syntactic priming: English L2 speakers’ production of dative constructions. Studies in Second Language Acquisition.

[CR51] Meisel JM, Clahsen H, Pienemann M (1981). On determining developmental stages in natural second language acquisition. Studies in Second Language Acquisition.

[CR52] Metz C (2016). Forget apple vs. the FBI: WhatsApp just switched on encryption for a billion people.

[CR53] Milrad M, Jackson M (2008). Designing and implementing educational mobile services in university classrooms using smart phones and cellular networks. International Journal of Engineering Education.

[CR54] Mohammadi MO, Jabbari AA, Fazilatfar A (2018). The impact of the asynchronous online discussion forum on the Iranian EFL students’ writing ability and attitudes. Applied Research on English Language.

[CR55] Oxfordenglishtesting.com (2020). What is the Oxford Online Placement Test?.

[CR56] Pearson WS (2020). Research article titles in written feedback on English as a second language writing. Scientometrics.

[CR57] Petersen S, Divitini M, Milrad M, Hoppe H, Kinshuk (2005). Language learning: From individual learners to communities. IEEE international workshop on wireless and mobile technologies in education.

[CR58] Pourmousavi Z, Mohamadi Zenouzagh Z (2020). A comparative study of the effect of teacher’s group and individual feedback on Iranian EFL learners’ learning of speech acts in apology letter writing. Asian-Pacific Journal of Second and Foreign Language Education.

[CR59] Rahimi Domakani M, Roohani A, Abdollahian Z (2010). The effect of direct and indirect written corrective feedback on grammatical collocations in L2writing. The Journal of Teaching Language and Literature Society of Iran.

[CR60] Rassaei E (2017). Video chat vs. face-to-face recasts, learners’ interpretations and L2 development: A case of Persian EFL learners. Computer-Assisted Language Learning.

[CR61] Rassaei E, Moeinzadeh A, Youhannaee M (2012). Effects of recasts and metalinguistic corrective feedback on the acquisition of implicit and explicit L2 knowledge. Journal of Language Teaching and Learning.

[CR62] Robb T, Ross S, Shortreed I (1986). Salience of feedback on error and its effect on EFL writing quality. TESOL Quarterly.

[CR63] Schiffrin D (1987). Discourse markers.

[CR64] Semke HD (1984). Effects of the red pen. Foreign Language Annals.

[CR65] Sheen Y (2007). The effect of focused written corrective feedback and language aptitude on ESL learners’ acquisition of articles. TESOL Quarterly.

[CR66] Stockwell G (2010). Using mobile phones for vocabulary activities: Examining the effect of the platform. Language Learning & Technology.

[CR67] Storch N, Wigglesworth G (2010). Learners’ processing, uptake, and retention of corrective feedback on writing: Case studies. Studies in Second Language Acquisition.

[CR68] Truscott J (1996). The case against grammar correction in L2 writing classes. Language Learning.

[CR69] Truscott J (1999). The case for “the case for grammar correction in L2 writing classes”: A response to Ferris. Journal of Second Language Writing.

[CR70] Truscott J (2007). The effect of error correction on learners’ ability to write accurately. Journal of Second Language Writing.

[CR71] Truscott J, Hsu AYP (2008). Error correction, revision, and learning. Journal of Second Language Writing.

[CR72] Van Beuningen C, de Jong NH, Kuiken F (2012). Evidence on the effectiveness of comprehensive error correction in Dutch multilingual classrooms. Language Learning.

[CR73] Vickov G, Jakupcevic E (2017). Discourse markers in non-native EFL teacher talk. Studies in Second Language Learning and Teaching.

[CR74] Walker, R. (2013). “I don’t think I would be where I am right now”. Pupil perspectives on using mobile devices for learning. *Research in Learning Technology*, *21*. 10.3402/rlt.v21i0.22116.

[CR75] Yeha S, Lob J (2009). Using online annotations to support error correction and corrective feedback. Computers & Education.

